# Private Sector Participation and Health System Performance in Sub-Saharan Africa

**DOI:** 10.1371/journal.pone.0013243

**Published:** 2010-10-07

**Authors:** Joanne Yoong, Nicholas Burger, Connor Spreng, Neeraj Sood

**Affiliations:** 1 RAND Corporation, Arlington, Virginia, United States of America; 2 Investment Climate Department, World Bank, Washington, D. C., United States of America; 3 University of Southern California and RAND Corporation, Los Angeles, California, United States of America; Kenya Medical Research Institute, Kenya

## Abstract

**Background:**

The role of the private health sector in developing countries remains a much-debated and contentious issue. Critics argue that the high prices charged in the private sector limits the use of health care among the poorest, consequently reducing access and equity in the use of health care. Supporters argue that increased private sector participation might improve access and equity by bringing in much needed resources for health care and by allowing governments to increase focus on underserved populations. However, little empirical exists for or against either side of this debate.

**Methodology/Principal Findings:**

We examine the association between private sector participation and self-reported measures of utilization and equity in deliveries and treatment of childhood respiratory disease using regression analysis, across a sample of nationally-representative Demographic and Health Surveys from 34 SSA economies. We also examine the correlation between private sector participation and key background factors (socioeconomic development, business environment and governance) and use multivariate regression to control for potential confounders. Private sector participation is positively associated with greater overall access and reduced disparities between rich and poor as well as urban and rural populations. The positive association between private sector participation and improved health system performance is robust to controlling for confounders including per capita income and maternal education. Private sector participation is positively correlated with measures of socio-economic development and favorable business environment.

**Conclusions/Significance:**

Greater participation is associated with favorable intermediate outcomes in terms of access and equity. While these results do not establish a causal link between private sector participation and health system performance, they suggest that there is no deleterious link between private sector participation and health system performance in SSA.

## Introduction

As the deadline for the Millennium Development Goals draws near, the aim of achieving universal and equitable access to quality health care remains in sharp contrast to the reality of daunting and persistent service gaps across the world. Since the 1980s, a number of international organizations and donors have started to work with the private health sector in developing countries, where the private health sector comprises all providers who exist outside the public sector, whether their aim is philanthropic or commercial [1].

However, support for the private sector in health care remains a much-debated and contentious issue. Critics argue that while consumers prefer the private sector due to perceived quality, easier access, and greater responsiveness, in many cases, the care provided in under-regulated developing country settings is of poor quality, with potential adverse implications for individual health outcomes as well as population disease control and drug resistance [2]–[7]. Others are concerned about user fees associated with private health services. They suggest that increasing the role of the private sector limits the use of health care among the poorest, who cannot afford to pay, consequently reducing access and equity in the use of health care [1], [8]–[9].

On the other hand, proponents of a greater role for the private health sector argue that given the resource-constraints of existing health systems, a more realistic approach to improving access to care is to acknowledge and build upon the opportunities and resources of an already operational private health sector [10]–[12]. Greater private sector participation might also improve equity by allowing governments and the public health system to focus on the poor and underserved.

While some interventions designed to improve utilization and equity through private for-profit sector engagement have been shown to be successful [13], overall, the lack of a robust evidence base has been a serious obstacle to analyzing the appropriate role of the private sector. This paper seeks to inform this debate by documenting the association between the size of the private sector and health system performance in terms of access to health care and equity in health care use. In particular, we use a nationally-representative data on maternal and child health care use from 34 Sub-Saharan African (SSA) economies to examine the association between private sector participation and health system performance in terms of access to health care and equity in health care use.

We demonstrate that, in Sub-Saharan Africa, private-sector participation is positively and significantly associated with better health system performance, both in terms of access and equity. We find a strong positive association between increased private sector participation and access to health care facilities for births and treatment of acute respiratory illness (ARI). We also find a strong positive association between private sector participation and equity measures – private sector participation is positively associated with reduced urban-rural and rich-poor disparities in access to health care facilities for births and treatment of ARI. These relationships are robust to the introduction of controls, including per capita GDP and maternal education.

## Methods

### Data

We use data from the Demographic and Health Surveys (DHS) from all SSA countries from which data are publicly available from 1994 to the present (Table 1) for this analysis (34 countries). The Standard DHS are nationally-representative household surveys conducted periodically by Measure DHS in several developing countries. In countries where multiple rounds of data collection have been collected, we include only the most recent round of the Standard DHS data for each country. As of June 2010, Measure DHS has conducted 140 surveys in 41 Sub-Saharan African countries, including Standard DHS surveys as well as smaller specialized surveys. Data from 5 countries (Botswana, Cape Verde, Eritrea, Mauritania, Sao Tome and Principe) is currently not available. 2 countries were omitted because the data were judged to be from a time period excessively removed from the rest of the sample (Burundi, 1987; Sudan 1990).

**Table 1:** Demographic and Health Surveys (DHS) for Sub-Saharan Africa.

Country Name	Year Completed	Sample Size		Wealth index data
		Primary respondents: Women 15–49	Children under 3 years of age	
Angola	2006	2,973	665	Yes
Benin	2006	17,794	9,773	Yes
Burkina Faso	2003	12,477	6,207	Yes
Cameroon	2004	10,656	4,928	Yes
Central African Rep.	1995	5,884	2,816	No
Chad	2004	6,085	3,316	Yes
Comoros	1996	3,050	1,145	No
Congo, Dem. Rep.	2007	9,995	5,519	Yes
Congo, Rep.	2005	7,051	3,065	Yes
Cote d'Ivoire	1999	3,040	1,258	No
Ethiopia	2005	14,070	5,765	Yes
Gabon	2000	6,183	2,741	No
Ghana	2008	4,916	1,826	Yes
Guinea	2005	7,954	3,943	Yes
Kenya	2009	8,444	3,733	Yes
Lesotho	2004	7,095	2,297	Yes
Liberia	2007	7,092	3,476	Yes
Madagascar	2009	17,375	7,415	Yes
Malawi	2004	11,698	6,799	Yes
Mali	2006	14,583	8,574	Yes
Mozambique	2003	12,418	6,177	Yes
Namibia	2007	9,804	3,244	Yes
Niger	2006	9,223	5,598	Yes
Nigeria	2008	33,385	17,215	Yes
Rwanda	2005	11,321	5,497	Yes
Senegal	2005	14,602	6,880	Yes
Sierra Leone	2008	7,374	3,533	Yes
South Africa	1998	11,735	3,119	No
Swaziland	2007	4,987	1,744	Yes
Tanzania	2005	10,329	5,290	Yes
Togo	1998	8,569	4,168	No
Uganda	2006	8,531	5,062	Yes
Zambia	2007	7,146	3,984	Yes
Zimbabwe	2006	8,907	3,217	Yes

Standard DHS surveys include household demographic information as well as information from women of reproductive age on maternal and child health indicators relevant to themselves and their children, including antenatal care, delivery care and treatment of childhood illness.

For all countries in the sample, we compute standardized population-representative measures, applying the DHS sample weights. We focus on births/deliveries and treatment of acute respiratory illness symptoms (ARI) for children under 3 years of age at the time of survey. Our two main outcome measures for access to health facilities are shown in Table 2: (1) the percentage of live births during the three years prior to the survey date that took place in a health facility, and (2) the percentage of children under 3 years of age that were treated at a health facility, of those who reported coughing and rapid breathing in the two weeks prior to the survey.

**Table 2:** Sample Summary Statistics.

	Mean	25^th^ percentile	75^th^ percentile	*N*
**Outcomes**				
***Access***				
% of deliveries in facility	50.3	38.9	63.6	*34*
% of children with ARI treated in facility	53.1	41.7	63.8	*33*
***Disparities***				
% of deliveries in facility: rich/poor	5.7	2.2	4.3	*28*
% of deliveries in facility: urban/rural	2.8	1.7	2.5	*34*
% of children with ARI treated at facility: rich/poor	1.8	1.4	2.1	*27*
% of children with ARI treated at facility: urban/rural	1.5	1.1	1.6	*33*
**Private Sector Participation**				
% of deliveries in private facility	7.7	1.6	12.1	*34*
% of children with ARI treated at private facility	17.4	8.7	23.0	*34*
				

For births, health facilities include public, nonprofit/NGO and mission/religious hospitals, clinics and health centers, but exclude in-home deliveries with traditional birth attendants. For ARI treatment, health facilities include all institutions defined by Measure DHS as the medical private sector, including public, nonprofit/NGO and mission/religious hospitals, clinics, health centers, dispensaries and pharmacies but excluding shops and traditional healers. Following Measure DHS convention, missing values are assumed to indicate no visit to that type of facility.

We also use these outcome measures to examine inequity in access to health care facilities. For births, the urban-rural frequency ratio is obtained by dividing the percentage of deliveries taking place in a health facility in urban households by the same percentage in rural households. Similarly, the rich-poor frequency ratio is obtained by dividing the percentage of deliveries taking place in a health facility in the highest wealth quintile by the same percentage in the lowest wealth quintile. The rich-poor frequency ratio is computable only for the subset of countries in which DHS provides a household wealth index as noted in Table 1. Equivalent frequency ratios are also computed for the ARI treatment measures.

Summary statistics for the key outcome variables are shown in Table 2. Overall, the data show relatively poor access to health care in SSA. On average, about half of all live births take place in a health facility and just over half of all children with ARI symptoms receive treatment in a health care facility. Table 2 also documents significant disparities in access to care across rich and poor households and urban and rural households. For example, on average rich households compared to poor households are 5.7 times more likely to give birth in a facility. Similarly, urban households are 2.8 times more likely to give birth in a facility. Smaller, but yet significant disparities are also observed in access to treatment for ARI. Finally, we also observe significant cross country variation in access and disparities in health care use.

To measure private sector participation, we computed the percentage of live births that took place in a private (for-profit or non-profit/mission) health facility and the percentage of children with ARI symptoms who were treated at a private health facility. Table 2 shows that less than one in ten births occur in private health care facilities and about one in five children with ARI symptoms received treatment at a private health facility. However, there is significant cross country variation in private sector participation. We examine the extent to which this variation is related to access and disparities in health care use.

To examine the correlates of private sector participation we obtain information on education, per capita income, the business environment, and governance from the DHS and other sources. We obtained information on maternal education from the DHS, GDP per capita from the World Development Indicators, business environment indicators from the World Bank 2010 Doing Business Report, and governance indicators on the regulatory environment from the World Bank's Country Policy and Institutional Assessment (CPIA) report. Information on the data and methodology underlying these rankings are available from the Doing Business website (http://www.doingbusiness.org/EconomyRankings/accessed September 10, 2010) and the World Bank IDA Resource Allocation Index website, (http://go.worldbank.org/S2THWI1X60 accessed September 10, 2010) respectively.

### Empirical Models

We use the above data to examine the association between private sector participation and access to health care facilities for births and treatment of ARI. We also examine the association between private sector participation and urban-rural and rich-poor disparities in access to health care facilities for these conditions. We start by estimating univariate regressions using ordinary least squares. The dependent variables are our measures of access or inequity and the independent variables are the corresponding measures of private sector participation. We report the magnitude of the coefficient and their statistical significance. We also test the robustness of results to exclusion of outliers from the analysis.

Next we examine the correlation between private sector participation and confounders that might be correlated with both private sector participation and our measures of equity and access. We focus on maternal education and per capita income, two important confounders that are known to affect access to health care and health outcomes. We also examine the extent to which private sector participation is correlated with the business environment and governance, factors that might influence private sector participation but should be unrelated to our measures of equity and access (except through private sector participation). For these analyses, we divide our sample of countries into two roughly equal groups – those with “high” private sector participation and those with “low” private sector participation. We compare mean values of confounders and other contextual factors across these groups using pair wise t-tests.

Finally, we use multivariate regressions to test if the associations documented in the initial univariate analysis are robust to controlling for potential confounders. In particular, we re-estimate the univariate regression models with per capita GDP and maternal education as additional control variables.

## Results

**Figure 1:**
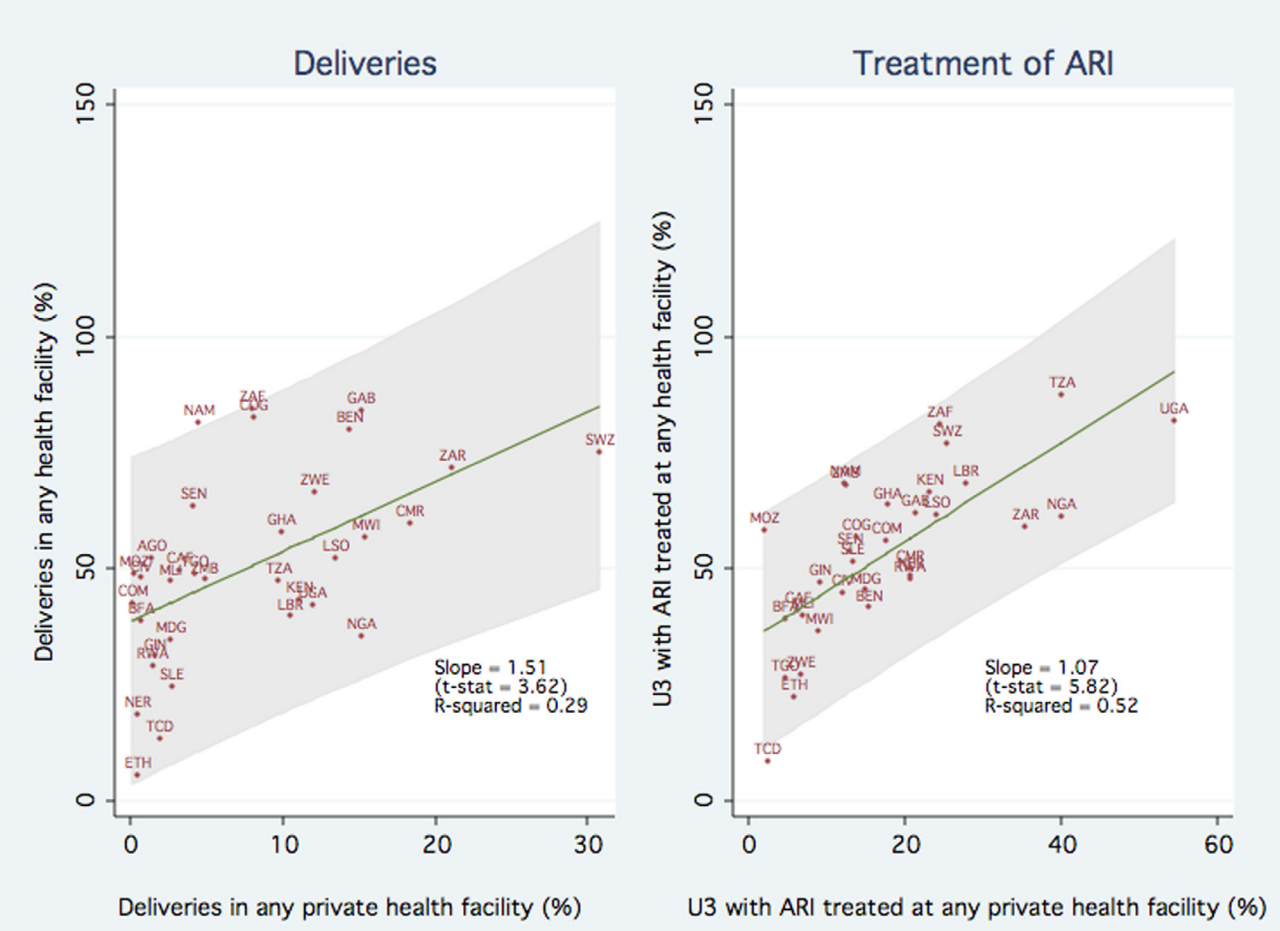
Association between Private Sector Participation and Access to Health Care Facilities.

Figure 1 shows the association between private sector participation and access to health care facilities, for births and ARI treatment. The left panel of Figure 1 plots the percentage of births in a health care facility (y-axis) as a function of the percentage of births in a private health facility (x-axis). The figure clearly shows that private sector participation is strongly associated with increased use of health care facilities. The estimated slope of the fitted regression line is 1.51 (p-value <0.01), indicating that a 10 percentage point increase in the proportion of children born in private facilities is associated with a 15.1 percentage point increase in the proportion of births taking place in any facility. To illustrate, moving from the 25^th^ percentile of private sector participation (1.6 percent of all births) to the 75^th^ percentile (12.1 percent of all births) is associated with a 165.6 percentage point increase in the proportion of births in any facility.

The right panel of Figure 1 plots the percentage of children with ARI symptoms taken to any facility (y-axis) against the percentage of children with ARI symptoms taken to a private sector facility (x-axis). The estimated slope of the fitted regression line is 1.07 (p-value <0.01), indicating a one for one relationship between ARI treatment at a private facility and ARI treatment at any facility. Overall the results in Figure 1 are consistent with the hypothesis that increased private sector participation improves access to health care and does not crowd out public sector participation in health care.

**Figure 2:**
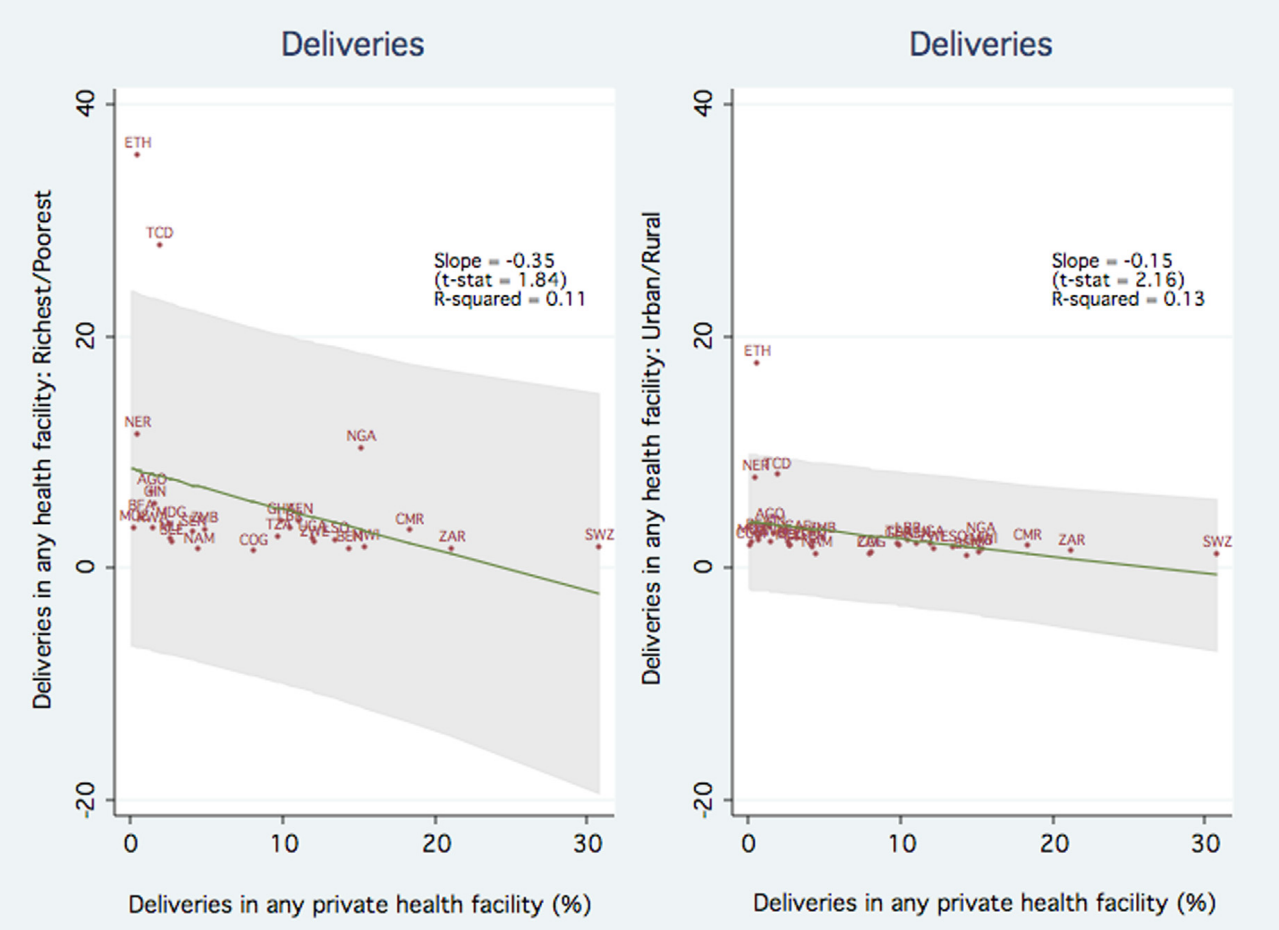
Association between Private Sector Participation and Equity in Use of Health Facilities for Deliveries.

We next examine the association between disparities in care and private sector participation. Figure 2 shows the association between private sector participation and inequity as measured by frequency ratios in use of health care facilities. The left panel of Figure 2 plots rich-poor disparities in facility births as a function of percentage of births in a private facility. The estimated slope of the fitted regression line is - 0.35 (p-value <0.10), demonstrating a negative association between private sector participation and rich-poor disparities. The results suggest that in a country at the 25^th^ percentile of private sector participation, women from rich households are 8.0 times more likely than those from poor households to give birth in a health care facility. In contrast, in a country at the 75^th^ percentile of private sector participation, women from rich households are only 4.4 times more likely than those from poor households to give birth in a facility. The right panel plots the frequency ratio for urban vs rural households on the percentage of institutional deliveries in the private sector. The estimated slope of the fitted regression line is - 0.15 (p-value <0.05). This result holds same implications as the prior analysis, showing that increased private sector participation is associated with a both a reduction in rich-poor and urban-rural disparities.

However, the scatter plots reveal that the estimates might be influenced by the presence of four significant outliers – Ethiopia, Chad, Niger and Nigeria. Therefore, we repeated the analysis by excluding these outliers. The estimated slope coefficients are reduced in magnitude but remain negative and increase in statistical significance (a coefficient of -0.08 and p-value <0.05 with the rich-poor frequency ratio as the dependent variable, and a coefficient of 0.08 and p-value <0.05 with the rich-poor frequency ratio as the dependent variable, and a coefficient of -0.05 with a p-value <0.01 with the urban-rural frequency ratio as the dependent variable). The implied effects on the inequality measure are correspondingly smaller: in a country at the 25^th^ percentile of private sector participation, women from rich households are 3.7 times more likely than poor households to give birth in a health care facility, while a country at the 75^th^ percentile of private sector participation, women from rich households are only 2.9 times more likely than poor households to give birth in a facility.

**Figure 3:**
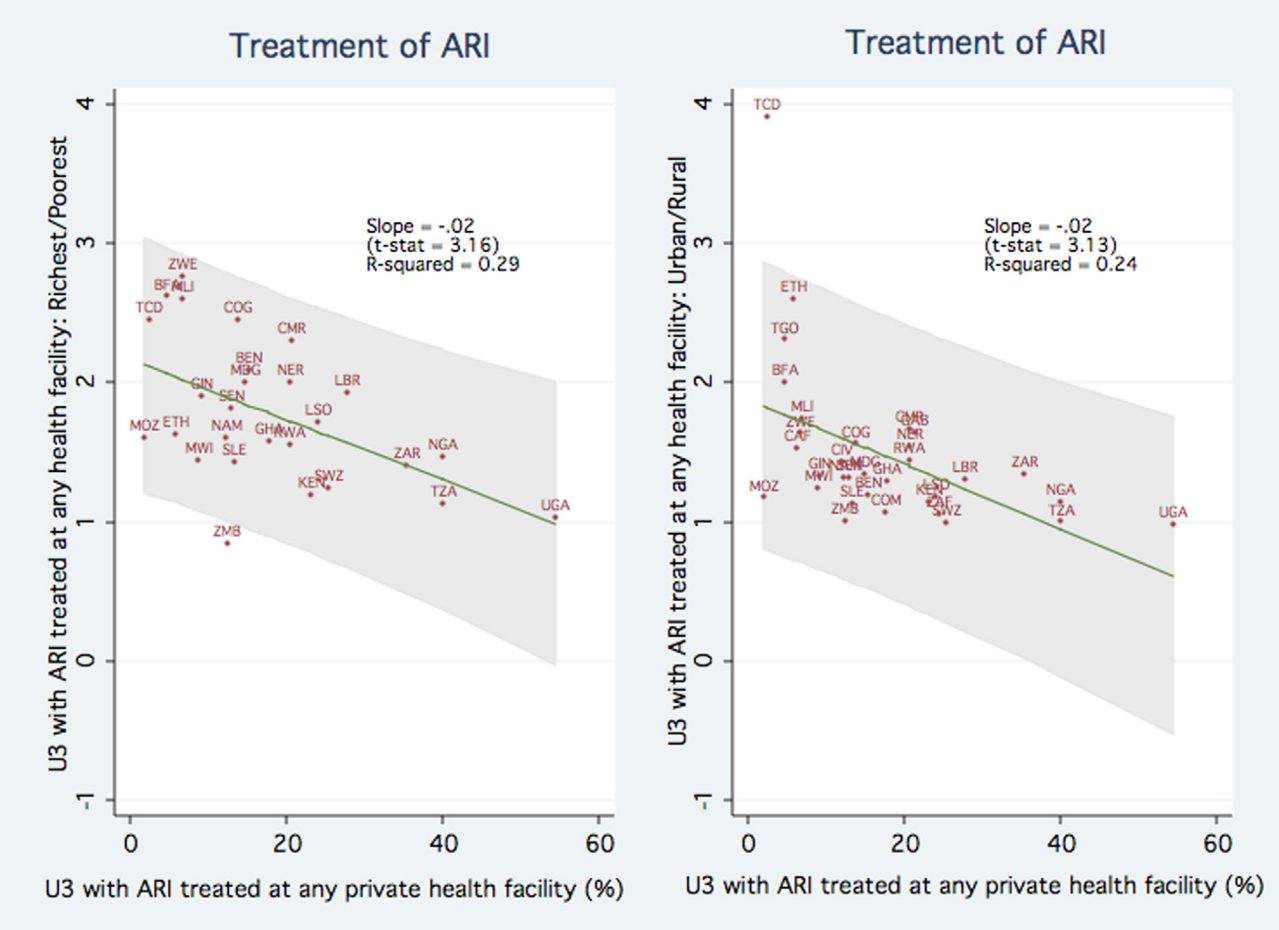
Association between Private Sector Participation and Equity in ARI Treatment.

We perform the analogous regressions of the frequency ratios for children with ARI symptoms receiving any treatment on the fraction of children with ARI symptoms receiving treatment in private sector facility. Figure 3 shows that for both the richest/poorest and urban/rural frequency ratios, the slope coefficients are approximately -0.02, and statistically significant (p-value <0.01). The results imply that in a country at the 25^th^ percentile of private sector participation for ARI treatment, children from rich households are 2.0 times more likely than poor households to be taken to a facility, while a country at the 75^th^ percentile of private sector participation, children from rich households are only 1.7 times more likely to be taken to a facility.

As an additional robustness check, we replicated the regression analysis using concentration indices [14] for delivery and ARI treatment by rural/urban sector and wealth quintile respectively as alternative outcome measures. In all cases, we find that the degree of private sector participation is negatively and significantly correlated with the concentration index, with p-values <0.05. Overall, the results suggest that higher private sector participation is associated with lower rich-poor and urban-rural disparities in access to health care facilities.

### Background Factors

In the analysis above, we find that greater private sector participation is positively associated with higher levels of service utilization and negatively associated with rich-poor and urban-rural disparities in health care access and use. However, higher private sector participation may be affected by other variables that also affect access and equity. Consistent with other research [15], Table 3 shows that, in addition to an increased level of overall service utilization, countries with a relatively large share of private sector participation tend to also have significantly higher levels of maternal education and also higher levels of GDP per capita, although differences in per capita income are not statistically significant. The previously estimated relationships may therefore be confounded by differences in socioeconomic development (particularly maternal education, a well-established key determinant of health service utilization and child health outcomes).

**Table 3:** Other Socioeconomic Indicators, Small vs. Large Private Sector.

	% live births in private sector		
Mean of outcome variables:	Smaller (Below Median)	Larger (Above Median)	T-test statistic for equality of means
Maternal education in years (a)	2.2	5.5	−5.33*
GDP per capita in survey year (USD at current exchange) (b)	735.5	1114.0	−1.03
Doing Business rank (c)	146	131	1.20
CPIA – Business Regulatory environment(d)	3.0	3.2	−0.49

	% children with ARI treated in private sector		
Mean of outcome variables:	Smaller (Below Median)	Larger (Above Median)	T-test statistic for equality of means
Maternal education in years (a)	3.0	4.8	−2.19*
GDP per capita in survey year(USD at current exchange) (b)	709.1	1046.6	−0.92
Doing Business rank (c)	145.7	129.3	1.25
CPIA – Business Regulatory environment(d)	3.0	3.3	−0.90

DHS survey data; (b) World Development Indicators; (c) Doing Business 2010; (d) CPIA 2008;

*p<0.05;

The last two rows of Table 3 show how countries with the highest and lowest shares of private sector participation vary in terms of their present Doing Business rank and CPIA business regulatory environment index. These measures capture regulations, policies and institutional environment that are likely to promote private sector participation, but affect health system outcomes only through changes in the private sector. Countries with greater private sector participation tend to have a higher Doing Business rank, although the difference is not statistically significant. These countries also are more likely to receive a higher score on the CPIA business regulation index, however the difference is not statistically significant.

### Multivariate Regressions

To control for potentially confounding background factors, we re-estimate the earlier regressions, adding controls for maternal education and the log of GDP per capita. Columns 1 and 2 in Table 4 show the results of the initial univariate regressions with service utilization as the outcome variables, while Columns 3 and 4 show the results with controls added.

**Table 4:** Overall Service Utilization, OLS Regression Estimates.

	(1)	(2)	(3)	(4)
	% deliveries in facility (SD)	% children with ARI symptoms taken to facility (SD)	% deliveries in facility (SD)	% children with ARI symptoms taken to facility (SD)
% deliveries in private facility	1.507* (0.416)		0.887+ (0.443)	
% U3 with ARI symptoms taken to private facility		1.067* (0.183)		0.920* (0.177)
Log GDP per capita (current USD)			8.241* (3.428)	4.660+ (2.718)
Average maternal education (years)			1.866 (1.596)	1.297 (1.076)
Constant	38.622* (4.385)	34.525* (3.857)	−16.126 (19.456)	2.722 (15.629)
R-squared	0.269	0.507	0.486	0.618
N	34	33	34	33

Note: Standard errors in parentheses.

+ p<0.10,

*p<0.05.

In both cases, the coefficients on the share of private sector service utilization show some attenuation, but the results are qualitatively unchanged and the statistical significance is robust. The value of both coefficients is close to 0.9. We also note that the coefficient on log GDP per capita is positive and significant, and the coefficient on maternal education is positive, as expected, but not statistically significant.

In Table 5 and 6 we perform the same analysis with the frequency ratios for deliveries and treatment of ARI symptoms respectively. Again, the coefficients on the share of private sector service utilization are reduced in magnitude, but the results are substantively and statistically unchanged.

**Table 5:** Frequency Ratios for Deliveries in Facility, OLS Regression Estimates.

	(1)	(2)	(3)	(4)
	Frequency ratio: Richest/Poorest	Frequency ratio: Urban/rural	Frequency ratio: Richest/Poorest	Frequency ratio: Urban/rural
% deliveries in private facility	−0.085* (0.031)	−0.046* (0.012)	−0.071+ (0.039)	−0.036* (0.015)
Log GDP per capita (current USD)			0.222 (0.322)	−0.067 (0.114)
Average maternal education (years)			−0.093 (0.141)	−0.049 (0.053)
Constant	3.830* (0.359)	2.398* (0.130)	2.705 (1.881)	2.943* (0.653)
R-squared	0.219	0.320	0.167	0.336
N	24	30	24	30

Note: Standard errors in parentheses.

+ p<0.10,

*p<0.05,

ETH,NER,TCD and NGA omitted.

**Table 6:** Frequency Ratios for Treatment of Children Under 3 with ARI Symptoms, OLS Regression Estimates.

	(1)	(2)	(3)	(4)
	Frequency ratio: Richest/Poorest	Frequency ratio: Urban/rural	Frequency ratio: Richest/Poorest	Frequency ratio: Urban/rural
% children with ARI symptoms taken to private facility	−0.022* (0.007)	−0.023* (0.007)	−0.019* (0.008)	−0.019* (0.008)
Log GDP per capita (current USD)			0.024 (0.131)	0.006 (0.125)
Average maternal education (years)			−0.035 (0.047)	−0.059 (0.050)
Constant	2.165* (0.151)	1.880* (0.157)	2.108* (0.761)	1.987* (0.719)
R-squared	0.257	0.216	0.214	0.220
N	27	33	27	33

Note: Standard errors in parentheses. + p<0.10,

*p<0.05.

### Limitations

The findings of these analyses should be viewed in light of its limitations. Firstly, while Measure DHS data provides the unique benefit of nationally representative and comparable data across several countries, some caveats should be borne in mind. Sample sizes vary but can be relatively small for some individual countries. Due to the variation in data-collection activities, the range of dates in the sample is large. Furthermore, there is some concern about measurement error in determining ownership type for health facilities. Especially the distinction between faith based not-for-profit facilities and government facilities might not be clear to some respondents. In other instances facilities might be run as public-private partnerships and respondent assignment of such facilities to a particular ownership type might be arbitrary. Finally, what constitutes a health facility is a subjective decision. As such, researchers may adopt different classification of providers, leading to different definitions of “health facilities”. For example, we classify pharmacies as health facilities and other researchers might disagree with this assumption.

However, in sensitivity analysis we find that our results are robust to most of these limitations of the DHS data. We find that the magnitude and statistical significance of our results are largely unchanged when: (1) restricting our sample to countries for which sample size is larger than 5000, (2) restricting analyses to data from surveys conducted after 2004, and (3) excluding pharmacies as health facilities.

Secondly, even though we control for two of the most likely potential confounders - maternal education and per capita income - the results might still be biased because of other confounders related to private sector participation and health system performance. For example, countries with better functioning transportation infrastructure (e.g., roads, ports) may have both greater private sector participation and better overall access. However, even as associations, these results suggest that there is no obvious, deleterious link between private sector participation and health care access and equity in SSA. The relationship between the amounts of care the private sector provides and the measured health care access population is consistently positive, and there is a similar positive association between private sector participation and various measure of equity in health care access

Finally, we focus on private sector participation in maternal and child health in SSA and the findings may therefore not extend to other health conditions or regions. Moreover, because we have data aggregated at the country level, the results might mask substantial within country heterogeneity in outcomes and private sector participation.

## Discussion

We find that in Sub-Saharan Africa, private sector participation in delivery and treatment of childhood respiratory disease is significantly correlated with greater overall access to these services and reduced disparities between rich and poor as well as urban and rural populations. These results are robust to controlling for per capita GDP and maternal education two important confounding factors that are correlated with both increased private sector participation and improved health care access.

Our findings provide new evidence for the debate about the appropriate role of the private health sector, as they show that greater participation of the private health sector is associated with favorable intermediate outcomes in terms of access to care and equity. However, it is important to note that we are unable to measure two critical and controversial facets of private facilities: the user fee charged at facilities and the technical quality of care provided. Evidence related to these in both the public and private sector is mixed. Several of the previously-cited studies have documented poor quality care in the private sector, but new evidence from recent multi country studies suggests that quality of care and provider competence is roughly equivalent in the public and private health sector [16]. With respect to user fees, some SSA countries continue to charge for services in public facilities, and there is no systematic evidence on whether user fees in the public sector are lower than in the private sector [17]. Another related caveat is that our measures of private sector participation reflect the proportion of the population using private health facilities, and cannot be generalized to cover the expansion of relatively unregulated non-facility based private sector such as drug peddlers and traditional healers.

Finally, although we provide new evidence on the appropriate role of the private health sector, several questions remain. Ultimately, there might be no single answer to this issue that is right for all countries. The appropriate role of the private sector might depend on the capacity of governments to provide effective stewardship and regulation, the health care financing environment, and the organization of the public health sector [18]. We hope that our study points to the potential importance of the private health sector in Sub-Saharan Africa and motivates further fact based research and discussion on this important policy area. In future research, and based on ongoing data collection on policies and regulations towards the private health sector across Africa, we hope to examine how public policy can improve the effectiveness of the private health sector in meeting broader national health goals.

## References

[1] Mills A, Brugha R, Hanson K, McPake B (2002). What can be done about the private health sector in low-income countries.. Bulleting of the World Health Organization.

[2] Brugha R, Zwi A (1998). Improving the quality of private sector delivery of public health services: challenges and strategies.. Health Policy and Planning.

[3] Sauerborn R (2001). Low quality of care in low income countries: is the private sector the answer? Int.. Journal for Quality in Health Care.

[4] Zwi A, Brugha R, Smith E (2001). Private health care in developing countries: If it is to work, it must start from what users need.. BMJ.

[5] Chakraborty S, Frick K (2002). Factors influencing private health providers' technical quality of care for acute respiratory infections among under-five children in rural West Bengal, India.. Social Science and Medicine.

[6] Kamat VR (2001). Private practitioners and their role in the resurgence of malaria in Mumbai (Bombay) and Navi Mumbai (New Bombay), India: serving the affected or aiding an epidemic?. Social Science and Medicine.

[7] Gilson L, Doherty J, Loewenson R, Francis V (2007). Challenging inequity through health systems.. Final report, Knowledge Network on Health Systems, WHO Commission on the Social Determinants of Health.

[8] Dahlgren G, Whitehead M (2007). A framework for assessing health systems from the public's perspective: the ALPS approach.. International Journal of Health Services.

[9] Oxfam (2009). Blind Optimism, Challenging the myths about private health care in poor countries.. Oxfam Briefing Paper.

[10] Berman, Peter (1998). Rethinking health care systems: private health care provision in India.. World Development.

[11] Preker A, Harding A (2003). Private Participation in Health Services.. World Bank.

[12] International Finance Corporation (2007). The Business of Health in Africa: Partnering with the Private Sector to Improve People's Lives..

[13] Patouillard E, Goodman C, Hanson K (2007). Can working with the private sector improve access of the poor to quality health services? A systematic review of the literature.. International Journal for Equity in Health.

[14] Kakwani N, Wagstaff A, van Doorslaer E (1997). Socioeconomic inequalities in health: Measurement, computation, and statisical inference.. Journal of Econometrics.

[15] Hanson K, Berman P (1998). Private health care providers in developing countries: a preliminary analysis of levels and composition.. Health Policy and Planning.

[16] Das J, Hammer J, Leonard K (2008). The quality of medical advice in low-income countries.. Journal of Economic Perspectives.

[17] World Health Organization (2008). The World Health Report 2008.. http://www.who.int/whr/2008/en/index.html.

[18] Hanson K, Gilson L, Goodman C, Mills A, Smith R (2008). Is Private Health Care the Answer to the Health Problems of the World's Poor?. PLoS Med.

